# Effect of Probiotic *Clostridium butyricum* NCTC 7423 Supernatant on Biofilm Formation and Gene Expression of *Bacteroides fragilis*

**DOI:** 10.4014/jmb.2001.01027

**Published:** 2020-02-10

**Authors:** Da-Seul Shin, Ki-Jong Rhee, Yong-Bin Eom

**Affiliations:** 1 Department of Medical Sciences, College of Medical Sciences, Soonchunhyang University, Asan 3538, Republic of Korea; 2 Department of Biomedical Laboratory Science, College of Health Sciences, Yonsei University at Wonju, Wonju 6493, Republic of Korea; 3 Department of Biomedical Laboratory Science, College of Medical Sciences, Soonchunhyang University, Asan 158, Republic of Korea

**Keywords:** *Bacteroides fragilis*, *Clostridium butyricum*, probiotics, enterotoxigenic *Bacteroides fragilis* (ETBF), biofilm

## Abstract

Enterotoxigenic *Bacteroides fragilis* (ETBF) is the main pathogen causing severe inflammatory diseases and colorectal cancer. Its biofilm plays a key role in the development of colorectal cancer. The objective of this study was to determine the antagonistic effects of cell-free supernatants (CFS) derived from *Clostridium butyricum* against the growth and biofilm of ETBF. Our data showed that *C. butyricum* CFS inhibited the growth of *B. fragilis* in planktonic culture. In addition, *C. butyricum* CFS exhibited an antibiofilm effect by inhibiting biofilm development, disassembling preformed biofilms and reducing the metabolic activity of cells in biofilms. Using confocal laser scanning microscopy, we found that *C. butyricum* CFS significantly suppressed the proteins and extracellular nucleic acids among the basic biofilm components. Furthermore, *C. butyricum* CFS significantly downregulated the expression of virulence- and efflux pump-related genes including *ompA* and *bmeB3* in *B. fragilis*. Our findings suggest that *C. butyricum* can be used as biotherapeutic agent by inhibiting the growth and biofilm of ETBF.

## Introduction

*Bacteroides fragilis*, an obligate anaerobe, constitutes 1% to 2% of the normal microorganisms in the intestines of a healthy human [1]. However, it is a major pathogen causing inflammatory bowel diseases (IBD) such as Crohn's disease and ulcerative colitis [2, 3]. The virulence factors of *B. fragilis* such as capsular polysaccharide, outer membrane proteins and enterotoxin known as fragilysin (Bft) are closely associated with biofilm formation and pathogenicity of *B. fragilis* [4]. Above all, enterotoxigenic *B. fragilis* (ETBF) secreting Bft not only causes severe inflammatory diseases, but also contributes to the development of colorectal cancer (CRC) [5]. Recent studies have reported that more than 60% of the biofilms formed by ETBF secreting Bft are frequently found in patients with severe inflammatory diseases [6]. In addition, CRC development is affected by spatial arrangement of bacterial communities in a high-dimensional structure, indicating that biofilm shows pro-carcinogenic activity and is essential for CRC initiation [7]. Therefore, it is essential to identify an effective therapeutic alternative that prevents severe inflammatory diseases or CRC by inhibiting the growth and biofilm of ETBF secreting Bft.

Bacteriotherapy using probiotics is a safe and promising approach [8]. A probiotic is defined as a live microorganism that provides health benefits when administered in appropriate amounts [9]. Probiotics have beneficial effects, such as maintaining intestinal homeostasis by regulating the host immune systems and suppressing the clustering of pathogens [10, 11]. *Clostridium butyricum* strains are probiotic bacteria that are used to prevent antibiotic-associated diarrhea [12]. *C. butyricum*, an obligate anaerobe, is a common human and animal gut commensal bacterium and accounts for 10-20% of all human stool samples by microbial culture. They secrete an abundance of short-chain fatty acids (SCFAs), mainly butyrate and acetate, which promote the proliferation of enterocytes [13] and accelerate regulatory T cell generation [14]. *C. butyricum* also possesses antifungal and antibacterial activities against *Candida albicans*, *Clostridium difficile*, enterotoxigenic *E. coli* (ETEC), *Vibrio* spp. and *Helicobacter pylori* [15-17]. Further, a recent study by Hayashi *et al*. revealed that *C. butyricum* represses acute experimental colitis in mice by stimulating intestinal interleukin-10 (IL-10)–producing macrophages [18].

Although studies have actively investigated probiotics, few studies have analyzed the inhibitory effects of *C. butyricum* on intestinal biofilm formation by pathogens. Especially, the effect of *C. butyricum* against the growth and biofilm of ETBF secreting Bft has yet to be reported. In order to investigate the effect and the possible application of metabolites formed by *C. butyricum*, this study evaluated the antagonistic activity of cell-free supernatant (CFS) extracted from *C. butyricum* against the growth, biofilm formation and gene expression of *B. fragilis*. Our work widens our intellectual horizon on the potential antibacterial and antibiofilm activities of CFS derived from *C. butyricum* in addition to its role as biotherapeutic agent against ETBF secreting Bft.

## Materials and Methods

### Organisms, Media and Growth Conditions

The wild-type enterotoxigenic *Bacteroides fragilis* 86-5443-2-2 (WT-ETBF; *bft-2*), a wild-type non-enterotoxigenic *Bacteroides fragilis* NCTC 9343 (WT-NTBF), and a recombinant strain transformed via insertion of *bft-2* gene into WT-NTBF (rETBF; *bft-2*) and *C. butyricum* NCTC 7423 (ATCC 19398), a nontoxigenic strain, were used in this study [19]. *B. fragilis* strains and *C. butyricum* NCTC 7423 were cultured in brain heart infusion (BHI; Difco, Becton-Dickinson and Company, USA) supplemented with 1% glucose, 0.1% hemin, 0.5% yeast extract, and 0.05% L-cystine (BHIS) and reinforced clostridial medium (RCM; Difco, Becton-Dickinson and Company), respectively, at 37°C under anaerobic conditions [20]. All stock cultures were stored in broth with 20% glycerol at -80°C until testing.

### Preparation of *C. butyricum* Cell-Free Supernatant

The *C. butyricum* cell-free supernatant (CFS) was prepared as described previously [21]. In brief, *C. butyricum* NCTC 7423 was incubated with the reinforced clostridial medium at 37°C for 24 h under anaerobic conditions. The culture was centrifuged at 3,000 rpm for 15 min and the CFS was filtered with 0.2 µm pore size syringe filter (Advantec, Japan). To determine whether the inhibitory activity may be affected by the bacteriocin-like inhibitory substances, the CFS was adjusted to pH 6.5 using 1N NaOH to exclude the effect of organic acid [22].

### *C. butyricum* CFS Susceptibility Testing on *B. fragilis* Planktonic Cells

To determine the antibacterial activity of CFS derived from *C. butyricum*, the broth microdilution method was used according to the Clinical and Laboratory Standards Institute guidelines [23], with a few modifications. Briefly, overnight cultures of *B. fragilis* strains were grown in BHIS broth. The bacterial suspension was adjusted to the final concentration of 1 × 10^6^ CFU/ml and inoculated into a 96-well microtiter plate (BD Falcon, USA) along with the *C. butyricum* CFS or neutralized CFS to a total volume of 200 µl. The *C. butyricum* CFS or neutralized CFS was serially diluted two-fold in BHIS broth. Overnight-cultured *B. fragilis* strains were set as control. Following inoculation, the 96-well microtiter plate was incubated anaerobically at 37°C for 24 h. At the end of the incubation, the growth of planktonic cells was assessed at 600 nm (A_600_) wavelength using a Multiskan GO plate reader (Thermo Fisher Scientific, USA).

### Effect of *C. butyricum* CFS on Biofilm Formation and Preformed Biofilms of *B. fragilis*

The biofilm formation assay was performed in a 96-well microtiter plate as described previously [24, 25], with some modifications. Overnight-cultured *B. fragilis* suspensions were diluted in BHIS broth to a final density of 1 × 10^6^ CFU/ml and dispensed into a 96-well microtiter plate. The 2-fold serial dilutions of *C. butyricum* CFS in BHIS broth were added to wells of a 96-well microtiter plate and incubated at 37°C for 24 h under anaerobic conditions.

To establish the preformed biofilms, a reference to *B. fragilis* were prepared in BHIS broth at a density of 1 × 10^6^ CFU/ml, added to a 96-well microtiter plate and incubated anaerobically at 37°C for 24 h. At the end of the incubation, the preformed biofilms were rinsed with 0.01 M phosphate-buffered saline (PBS; pH 7.4) to remove non-adherent cells. *C. butyricum* CFS was diluted two-fold in fresh BHIS broth and inoculated into the preformed biofilms. A 96-well microtiter plate was incubated at 37°C for an additional 24 h under anaerobic conditions. The control was set as mentioned above. The inhibitory effect of *C. butyricum* CFS on biofilm formation and preformed biofilms of *B. fragilis* was quantitatively evaluated using the crystal violet assay as described below.

### Biofilm Quantitation Via Crystal Violet Assay

The crystal violet assay was performed to evaluate the biofilm biomass quantitatively as described previously [25, 26], with minor modifications. After the biofilm formation, the medium was aspirated and the biofilm was washed with PBS to remove unattached cells from wells. The 96-well microtiter plate was dried at 60°C for 1 h. The remaining biofilms were stained with 1% crystal violet for 5 min and rinsed with sterile distilled water to remove the dye. After drying at 60°C for 1 h, the crystal violet was extracted from the stained biofilms using 33% (v/v) acetic acid. The biofilm biomass was quantitatively determined by measuring absorbance at 570 nm (A_570_).

### Biofilm Metabolic Activity - XTT Reduction Assay

The metabolic activity of *B. fragilis* biofilms was analyzed using the XTT [2,3-bis(2-methoxy-4-nitro-5-sulfophenyl)-2H-tetrazolium-5-carboxanilide] reduction assay as described previously [27], with slight modifications. Briefly, the 96-well microtiter plate was used to establish the biofilms of three *B. fragilis* strains as described above. After the preformed biofilms were washed with PBS, a fresh BHIS broth containing the 2-fold serial dilutions of *C. butyricum* CFS was dispensed into preformed biofilms and incubated at 37°C for 24 h in anaerobic environments. Subsequently, the preformed biofilms were washed with PBS. The metabolic activity of *B. fragilis* biofilms was determined using the XTT cell proliferation assay kit (ATCC, USA) in accordance with the manufacturer’s instructions. Prior to the experiment, the XTT reagent was mixed with the activation reagent at a ratio of 50:1 (v/v), and 50 µl of the XTT/activation was dispensed into the preformed biofilms and incubated at 37°C in the dark for 3 h. To analyze the metabolic activity of *B. fragilis* biofilms, the specific absorbances were calculated at a test wavelength of 475 nm (A_475_) and a reference wavelength of 650 nm (A_650_) [28]. According to the XTT cell proliferation assay protocol, the specific absorbance of the sample is expressed mathematically as follows; Specific absorbance = A_475_ (Test) – A_475_ (Blank) – A_650_ (Test).

### Confocal Laser Scanning Microscopy

Confocal laser scanning microscopy (CLSM) was conducted using biofilms of three *B. fragilis* strains developed on a tissue culture-treated 24-well glass bottom imaging plate (Eppendorf AG, Germany, Cat. no.: 0030741021) as reported previously [29], with some modifications. *B. fragilis* strains in BHIS were dispensed into the wells at a concentration of 1 × 10^6^ CFU/ml with 2-fold serial dilutions of *C. butyricum* CFS and incubated at 37°C for 24 h under anaerobic conditions. Overnight-cultured *B. fragilis* strains were employed as control. Following incubation, biofilms formed on the well were washed with PBS and fixed with 3.7%(v/v) formaldehyde for 1 h. The amino groups in the *B. fragilis* biofilms were visualized by staining with fluorescein isothiocyanate isomer I (FITC, 10 µg/µl; Sigma-Aldrich, Germany) for 1 h. Carbohydrates of biofilms were stained with Concanavalin A-Alexa Fluor 594 conjugate (Con A, 0.1 µg/µl; C-11253, Molecular Probes, USA) for 30 min. To monitor the extracellular nucleic acids, biofilms were stained with 4, 6-diamidino-2-phenylindoldihydrochloride (DAPI, 1 mg/l; Molecular Probes) for 45 min. After each step, the stained biofilms were washed with PBS to remove unbound staining solution. All steps were conducted in a dark room. The biofilms of *B. fragilis* strains were visualized at excitation wavelengths of 495, 590, and 358 nm for FITC, Con A and DAPI, respectively, using Zeiss LSM-710 confocal laser microscope (Carl Zeiss, USA) and were imaged using ZEN software (Carl Zeiss).

### RNA Extraction and Quantitative Real-Time Polymerase Chain Reaction (qRT-PCR)

To extract the total RNA of *B. fragilis* strains, 1 × 10^6^ CFU/ml of *B. fragilis* in BHIS was inoculated with *C. butyricum* CFS serially diluted 2-fold and incubated at 37°C for 24 h in anaerobic environments. At the end of incubation, the cells were collected by centrifuging each bacterial suspension at 25,000 ×*g* for 1 min at 4°C. The total RNA of *B. fragilis* strains was extracted and purified through NucleoSpin RNA mini Kit (Macherey-Nagel, Germany) in compliance with the manufacturer’s protocol. The concentration and purity of extracted RNA was assessed using BioDrop µLITE (BioDrop Ltd., UK), and 1 µg of template was reverse-transcribed into cDNA in a 20 µl reaction volume, using ReverTra Ace qPCR RT Master Mix with gDNA Remover (TOYOBO, Japan). The qRT-PCR was performed to investigate the relative gene expression of *B. fragilis* outer membrane protein (*ompA*) associated with virulence factor and RND-type efflux pump-related *bmeB3*. Power SYBR Green PCR Master Mix (Applied Biosystems, USA) was employed to analyze the PCR amplification products. The qRT-PCR was conducted using a StepOnePlus Real-Time PCR System (Applied Biosystems). Primer sequences used in qRT-PCR are shown in Table 1 [30-32]. *16S rRNA* was used as a housekeeping gene. The thermal cycling conditions of qRT-PCR were as follows: an initial denaturation at 95°C (10 min), followed by 40 cycles of denaturation at 95°C (15 sec), annealing at 58°C (1 min), and extension at 72°C (20 sec). The annealing stage was set at 57°C (30 sec) for the *ompA* gene and 55°C (1 min) for the *bmeB3* gene. The relative gene expression of target genes was normalized to *16S rRNA* gene and evaluated using the formula of 2^-ΔΔCT^.

### Statistical Analysis

All data are representative of three independent experiments, and the data were indicated as means ± standard deviations (SD). The results were analyzed via one-way analysis of variance (ANOVA) followed by Dunnett’s test to evaluate the significant differences between the treated groups and the control group. The results of qRT-PCR were analyzed using the Student’s *t*-test. All statistical analyses were conducted using the GraphPad Prism version 5 (GraphPad Software, USA). Statistical significance was considered at *p < 0.05, **p < 0.01 and ***p < 0.001.

## Results

### Antibacterial Activity of *C. butyricum* CFS on *B. fragilis* Planktonic Cells

**Fig. 1:**
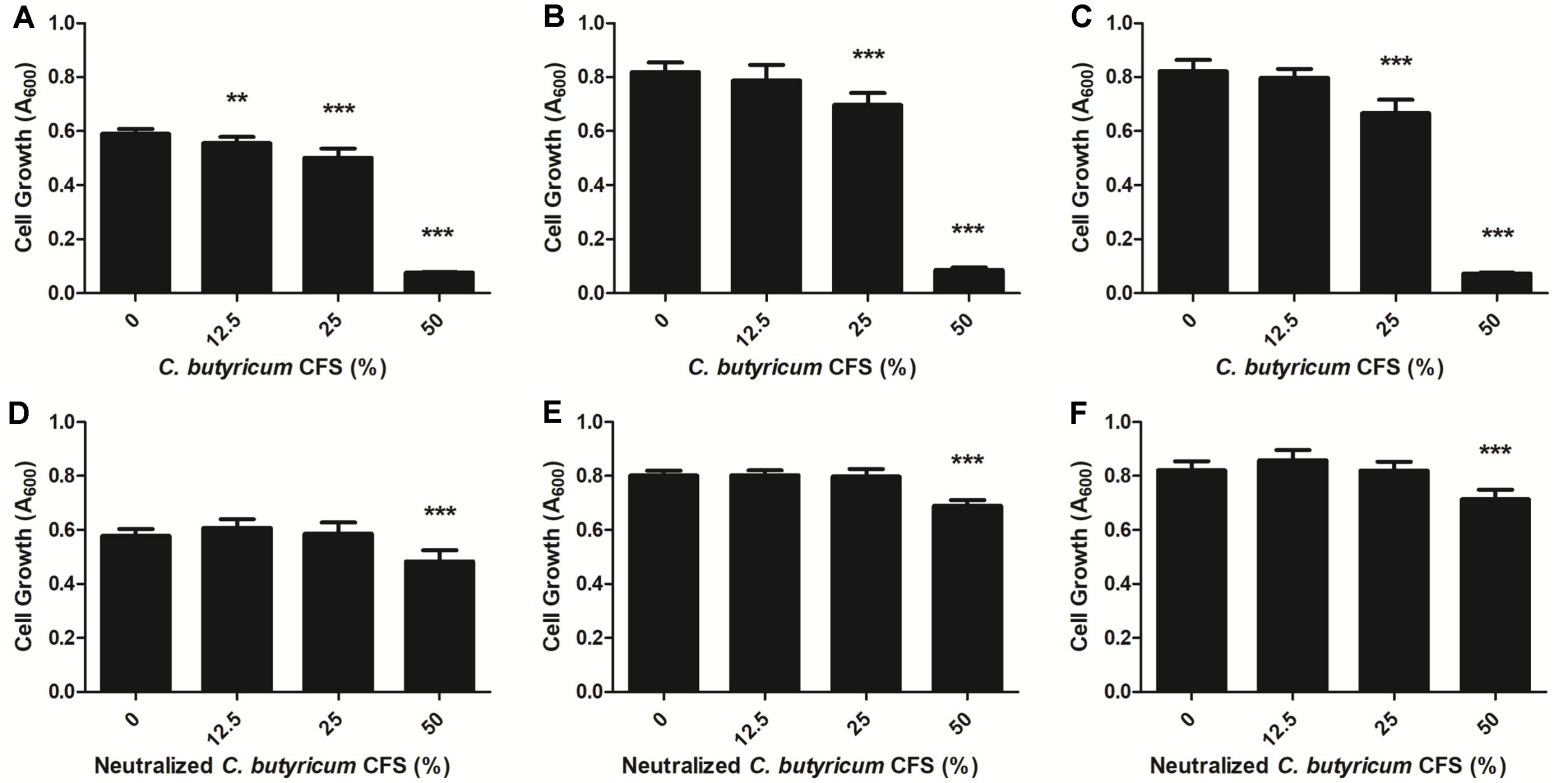
Antibacterial activity of *C. butyricum* CFS against the growth of *B. fragilis* planktonic cells. WT-ETBF (*bft-2*) (**A**, **D**), rETBF (*bft-2*) (**B**, **E**) and WT-NTBF (**C**, **F**) were incubated in the presence of *C. butyricum* CFS (**A**-**C**) or neutralized *C. butyricum* CFS (**D**-**F**) at 37°C for 24 h under anaerobic conditions. The growth of *B. fragilis* planktonic cells was analyzed at A_600_ using microplate spectrophotometers. The results are expressed as means ± standard deviations (SD). ** and *** describe significant differences at *p* < 0.01 and *p* < 0.001, respectively.

The antibacterial activity of *C. butyricum* CFS against *B. fragilis* strains was determined by measuring growth inhibition. A 50% *C. butyricum* CFS completely inhibited the growth of WT-ETBF (*bft*-2), rETBF (*bft*-2) and WT-NTBF by 99.48%, 98.38% and 100%, respectively (Figs. 1A-1C). A 25% *C. butyricum* CFS has a lower potential than 50%, but it significantly reduced the growth of *B. fragilis* strains. However, the growth of rETBF (*bft*-2) and WT-NTBF was not inhibited by 12.5% *C. butyricum* CFS. *C. butyricum* suppresses the growth of other microorganisms by producing a bacteriocin-like inhibitory compound [33]. In addition, neutralized probiotic CFS shows a partial similar effect to bacteriocin [21]. Therefore, to evaluate the effect of neutralized *C. butyricum* on the antibacterial activity, the *C. butyricum* CFS was adjusted to pH 6.5 to exclude organic acids in CFS. With the treatment of 50% *C. butyricum* CFS, the growth inhibition of three *B. fragilis* strains showed a statistically significant difference (Figs. 1D-1F). However, treatment with the neutralized *C. butyricum* CFS yielded less antibacterial activity than that of *C. butyricum* CFS against *B. fragilis* (Fig. 1), suggesting that the bacteriocin in *C. butyricum* CFS does not affect its growth.

### Inhibitory Effect of *C. butyricum* CFS on *B. fragilis* Biofilm Formation

**Fig. 2:**
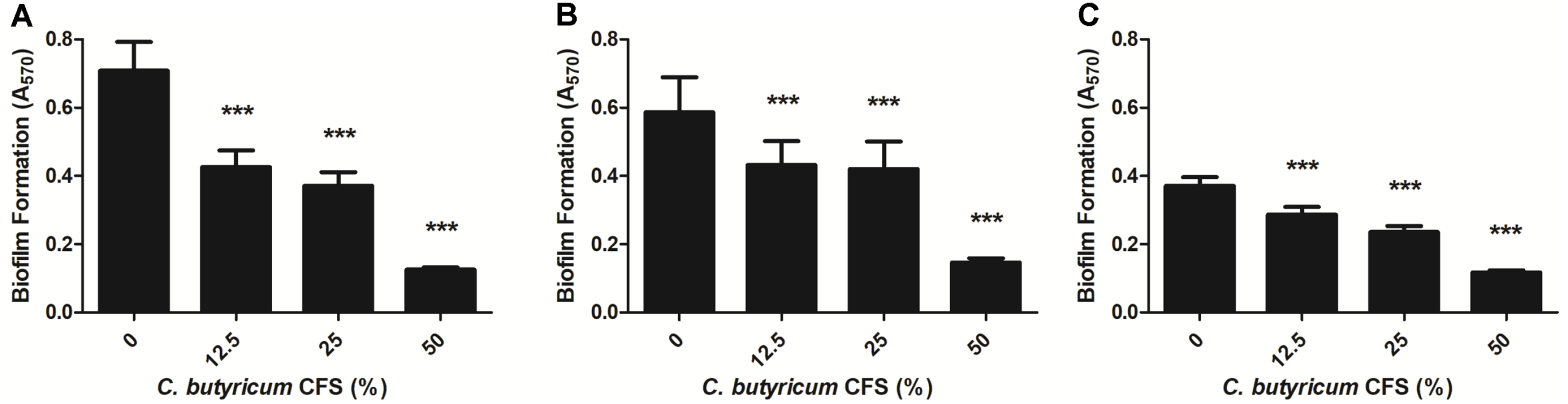
Inhibitory effect of *C. butyricum* CFS on biofilm formation of *B. fragilis*. WT-ETBF (*bft-2*) (**A**), rETBF (*bft-2*) (**B**) and WT-NTBF (**C**) were incubated with *C. butyricum* CFS at 37°C for 24 h under anaerobic conditions. Biofilms of *B. fragilis* strains were stained with 1% crystal violet and evaluated by measuring the absorbance at A_570_. The data are presented as means ± standard deviations (SD). ***p < 0.001 means statistical significance compared with the control group.

To determine the inhibitory effect of *C. butyricum* CFS on the biofilm formation of three *B. fragilis* strains, the biofilm was quantified by staining with crystal violet and measuring its absorbance at 570 nm (A_570_) wavelength. The biofilm formation of all strains used in this study was reduced by the *C. butyricum* CFS in a dose-dependent manner. More specifically, the biofilm formation of WT-ETBF (*bft*-2) was inhibited by 46.46%, 55.51% and 95.89% in the presence of 12.5%, 25%, and 50% *C. butyricum* CFS, respectively (Fig. 2A). The rETBF (*bft-2*) was also suppressed by 32.86%, 35.29%, and 93.48% (Fig. 2B) and WT-NTBF was inhibited by 30.16%, 48.61%, and 90.5% (Fig. 2C) following exposure to 12.5, 25, and 50% *C. butyricum* CFS, respectively. The results showed that the biofilm formation of all *B. fragilis* strains was inhibited by more than 90% by treatment with 50% *C. butyricum* CFS.

### Effect of *C. butyricum* CFS on Preformed Biofilms of *B. fragilis*

**Fig. 3:**
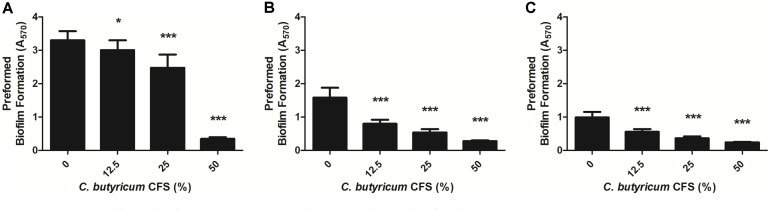
Eradicative effect of *C. butyricum* CFS on preformed biofilms of *B. fragilis*. The preformed biofilms of WT-ETBF (*bft-2*) (**A**), rETBF (*bft-2*) (**B**), and WT-NTBF (**C**) were incubated anaerobically at 37°C for 24 h in the presence of *C. butyricum* CFS. Biofilms of *B. fragilis* strains were stained with 1% crystal violet and determined by measuring at A_570_. The data are presented as means ± standard deviations (SD). *p < 0.05 and ***p < 0.001 indicate statistical significance compared with the control group.

As shown above, the *C. butyricum* CFS not only inhibited the biofilm formation of *B. fragilis* strains, but also potentially eliminated such preformed biofilms. In the presence of 12.5 to 25% *C. butyricum* CFS, the preformed biofilms by WT-ETBF (*bft*-2), rETBF (*bft*-2), and WT-NTBF were eradicated by 9.38 to 26.11%, 54.22 to 72.05%, and 49.28 to 71.9%, respectively (Fig. 3). Although the preformed biofilms of WT-ETBF (*bft*-2) were less affected than rETBF (*bft*-2) and WT-NTBF upon treatment with 12.5 to 25% *C. butyricum* CFS, the preformed biofilms of WT-ETBF (*bft*-2) were significantly eliminated by 93.24% upon treatment with 50% *C. butyricum* CFS (Fig. 3A). The rETBF (*bft*-2) was also eliminated by 90.19% at the same concentration (Fig. 3B). However, only 85% of the WT-NTBF preformed biofilms were eliminated by treatment with 50% *C. butyricum* CFS (Fig. 3C).

### Inhibitory Effect of *C. butyricum* CFS on the Metabolic Activity of Established *B. fragilis* Biofilms: XTT Reduction Assay

**Fig. 4:**
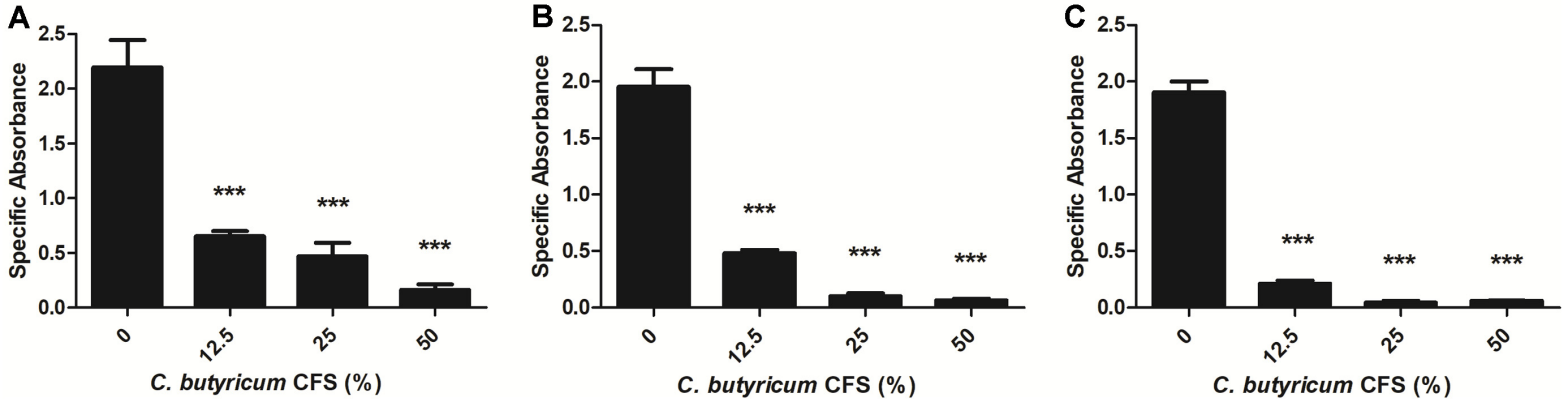
Inhibitory effect of *C. butyricum* CFS on the metabolic activity of established biofilms. The established biofilms of WT-ETBF (*bft-2*) (**A**), rETBF (*bft-2*) (**B**), and WT-NTBF (**C**) were incubated anaerobically at 37°C for 24 h, along with *C. butyricum* CFS. The metabolic activity of biofilm cells was assessed using XTT reduction assay. Specific absorbance was calculated as A_475_ (Test) – A_475_ (Blank) – A_650_ (Test). The results are presented as means ± standard deviations (SD). Asterisks (***) signify *p* < 0.05 compared with the control group.

A colorimetric XTT reduction assay was performed to determine the viability of *B. fragilis* cells within the biofilms in the presence of *C. butyricum* CFS. The specific absorbance was calculated using an XTT reduction assay. Even though the viability of WT-ETBF (*bft*-2) biofilm was less than 78.65% compared with other strains upon treatment with 25% *C. butyricum* CFS, the viability of all *B. fragilis* strains used in this study declined by more than 90% with 50% *C. butyricum* CFS (Fig. 4). As shown in Fig. 4, the metabolic activities of all strains were significantly reduced by treatment with the *C. butyricum* CFS in a dose-dependent manner. The results suggest that treatment with *C. butyricum* CFS suppresses the viability of *B. fragilis* cells within the biofilms as well as inhibits and eliminates *B. fragilis* biofilms.

### Confocal Laser Scanning Microscopy

**Fig. 5:**
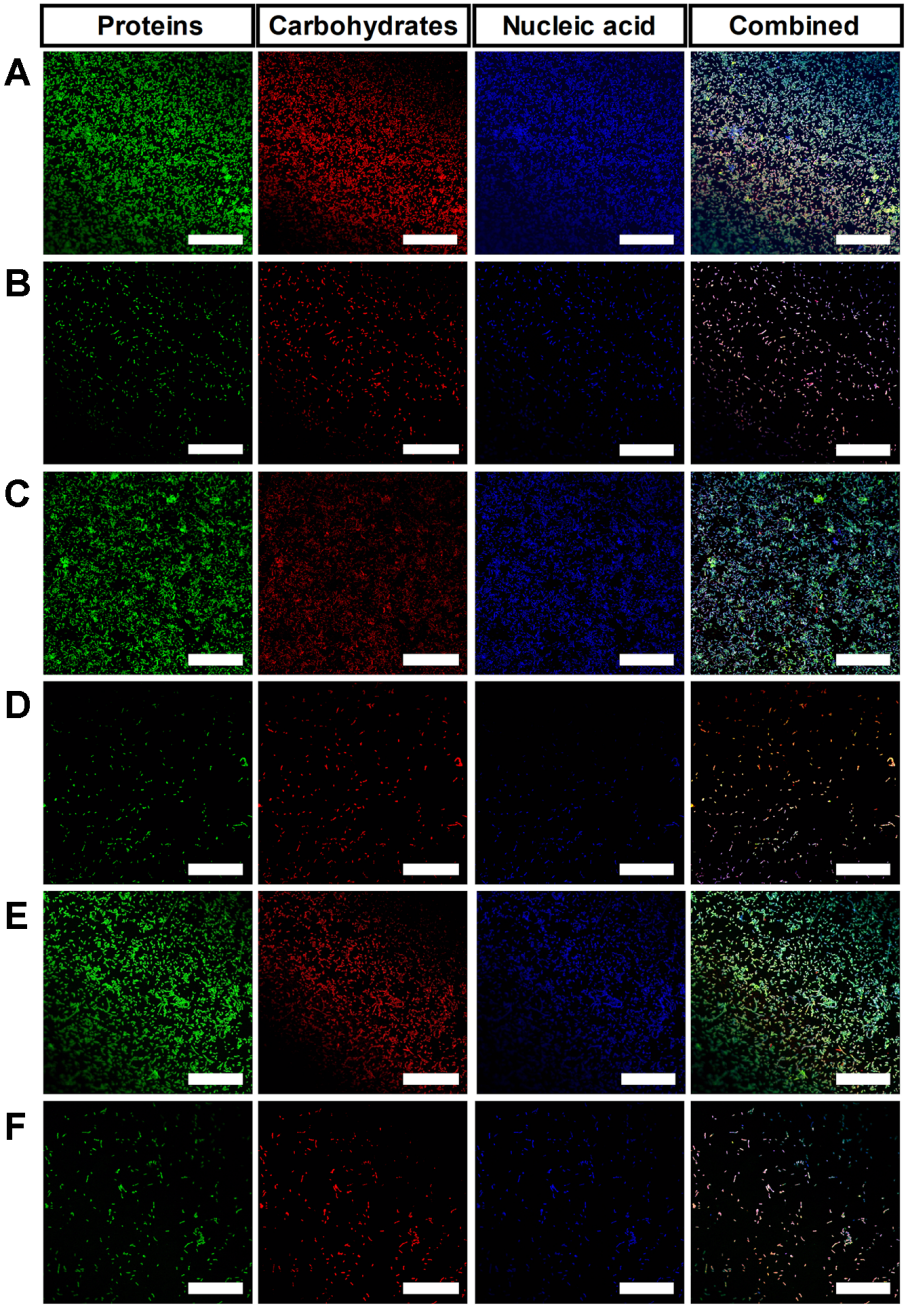
Confocal laser scanning microscopy (CLSM) images of *B. fragilis* biofilms. Proteins, carbohydrates, and nucleic acids occurring in *B. fragilis* biofilms were stained with FITC (Green), Con A (Red), and DAPI (Blue), respectively. WT-ETBF (*bft-2*) control (**A**) and after treatment with *C. butyricum* CFS (**B**). rETBF (*bft-2*) control (**C**) and after treatment with *C. butyricum* CFS (**D**). WT-NTBF control group (**E**) and after treatment with *C. butyricum* CFS (**F**). Biofilms were examined at 40 × magnification. The scale bar indicates 50 μm.

The extracellular polymeric substances (EPS) of biofilm matrix are mainly composed of proteins, carbohydrates and extracellular DNAs [34]. To visualize the inhibitory effect of *C. butyricum* CSF on the biofilms of *B. fragilis* strains, CLSM was performed by staining biofilms with fluorescent dyes. In the absence of *C. butyricum* CFS, the biofilms of WT-ETBF (*bft*-2), rETBF (*bft*-2) and WT-NTBF showed a thick and compact extracellular matrix and robust bacterial growth, as shown in Figs. 5A, 5C, and 5E, respectively. In addition, bacterial cell aggregation was clearly observed in the biofilms of all strains. By contrast, biofilms of all strains treated with 25% *C. butyricum* CFS were dispersed and disassembled, and bacterial cells were scattered due to lack of structural components compared with the untreated group (Figs. 5B, 5D, and 5F). Proteins and extracellular nucleic acids in the biofilm were remarkably reduced after treatment with *C. butyricum* CFS. These results show that *C. butyricum* CFS alters the architecture of extracellular matrix and reduces the cell density, thickness, and biomass of biofilms.

### Effect of *C. butyricum* CFS on the Expression of Virulence-and Efflux Pump-Related Genes in *B. fragilis*

**Fig. 6:**
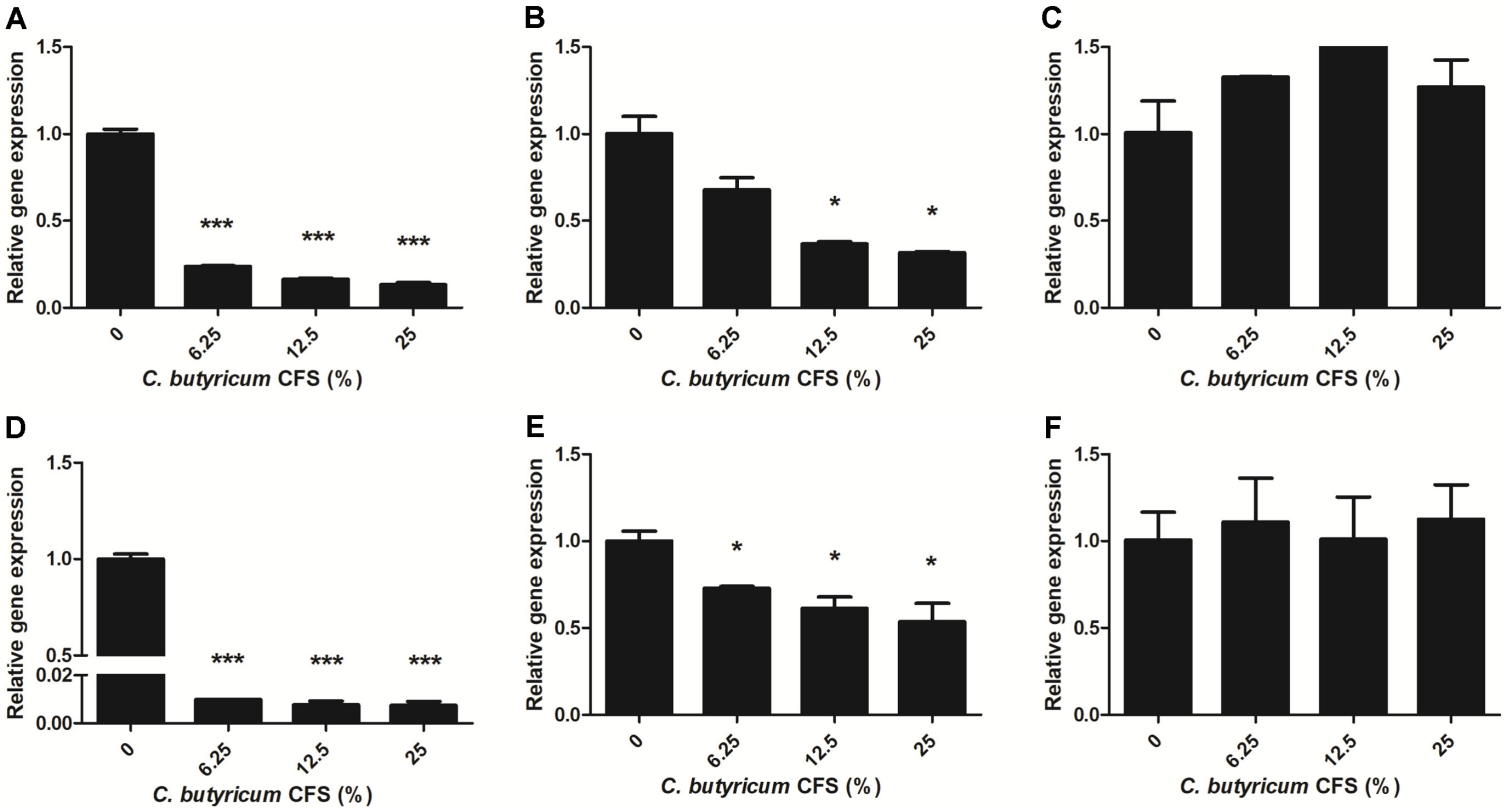
qRT-PCR analysis of *B. fragilis* outer membrane protein gene *ompA* and RND-type efflux pump-related gene *bmeB3*. *ompA* gene expression of WT-ETBF (*bft-2*) (**A**), rETBF (*bft-2*) (**B**), and WT-NTBF (**C**). *bmeB3* gene expression of WT-ETBF (*bft-2*) (**D**), rETBF (*bft-2*) (**E**) and WT-NTBF (**F**). The results are presented as fold changes relative to the control group. Student’s *t*-test was conducted to analyze the gene expression between treated and control groups. The results are presented as means ± standard deviations (SD). *p < 0.05 and ***p < 0.001 indicate statistical significance compared with the control group.

To obtain further insight into the molecular mechanism of *C. butyricum* CFS underlying the inhibition of *B. fragilis* biofilms, qRT-PCR analysis was performed. The expression of *ompA* and *bmeB3* genes in WT-ETBF (*bft*-2) and rETBF (*bft*-2) was considerably down-regulated by *C. butyricum* CFS in a concentration-dependent manner, whereas that of WT-NTBF was not affected (Fig. 6). Thus, exposure to 6.25%, 12.5%, and 25% concentrations of *C. butyricum* CFS downregulated the expression of *ompA* gene in WT-ETBF (*bft*-2) by 4.22-fold, 6.11-fold, and 7.57-fold, respectively (Fig. 6A), and reduced the expression of *ompA* gene in rETBF (*bft*-2) by 1.48-fold, 2.73-fold, and 3.17-fold, respectively (Fig. 6B). At similar concentrations, the expression of *bmeB3* gene in WT-ETBF (*bft*-2) was dramatically suppressed by 101.13-fold, 131.6-fold, and 136.82-fold, respectively (Fig. 6D), and the expression of *bmeB3* gene in rETBF (*bft*-2) was downregulated by 1.37-fold, 1.63-fold, and 1.87-fold, respectively (Fig. 6E). However, the levels of *ompA* and *bmeB3* gene in WT-NTBF were almost unaffected by the *C. butyricum* CFS, and the differences were not statistically significant (Figs. 6C and 6F).

## Discussion

Biofilms of ETBF carrying the *bft* gene are involved in the development of severe inflammatory diseases and CRC [7, 35]. Also, biofilm can lead to side effects such as selection of antibiotic-resistant bacteria and suppression of host immune system. For this reason, probiotics represent an alternative approach to preventing growth and biofilm of *B. fragilis* carrying the *bft* gene. *C. butyricum*, a probiotic, can selectively kill pathogens without affecting the normal intestinal flora [24] and possess anti-diabetic [36], antibacterial, antifungal [17] and anticancer effects [37]. However, the inhibition of *B. fragilis* by *C. butyricum* has yet to be reported. Therefore, our findings show that CFS extracted from *C. butyricum* significantly inhibits the growth and biofilm formation by ETBF carrying the *bft* gene.

The Bft toxin is a major virulence factor of *B. fragilis*. According to a study conducted by Pierce and Bernstein [38], ETBF carrying the *bft* gene was formed via mutations in non-toxigenic (NTBF) strain through evolutionary changes. Therefore, this study was conducted using WT-ETBF (*bft-2*), rETBF (*bft-2*), and WT-NTBF to confirm that CFS extracted from *C. butyricum* was strain-specific.

Meanwhile, *C. butyricum* produces bacteriocin-like inhibitory substances as well as organic acids, mainly butyrate. Bacteriocins are proteinaceous toxins produced by bacteria to inhibit the growth of similar or closely related bacterial strains [39]. Bacteriocin isolated from *C. butyricum* exhibited antimicrobial effects on diverse bacteria, but no effects on gram-negative bacteria [33]. Consistent with previous studies, our results also show that neutralized CFS, which has the same effect as bacteriocin, do not strongly inhibit the growth of *B. fragilis*.

HPLC analysis of Isono’s groups revealed that *C. butyricum* CFS contained 13.6 mmol butyrate, 8.3 mmol acetate, and 4.3 mmol formate [40]. Also, other studies confirmed that the amount of butyric acid among SCFAs increased most significantly when *C. butyricum* was inoculated on the in vivo models that caused various diseases [41, 42]. Butyrate, which accounts for most of the short-chain fatty acids (SCFAs) produced by *C. butyricum*, is soluble in water and has amphipathic actions [43]. Since 97% of the biofilm is composed of water, it is indicated that CFS (mostly butyrate) derived from *C. butyricum* can effectively pass throughout the biofilm [44, 45]. In addition, many studies have determined that butyric acid has an inhibitory effect on the growth and biofilm of various microorganisms such as *Trichosporon* spp., *Vibrio* spp., *Clostridium difficile* [45-47]. For these reasons, we expect that the action of the butyric acid is involved in the inhibitory effect of *C. butyricum* CFS against *B. fragilis* biofilm.

A previous study demonstrated that ETBF carrying the *bft* gene formed a large amount of biofilm compared with the non-toxigenic (NTBF) strain [38]. Similarly, our results also show that WT-ETBF (*bft-2*) and rETBF (*bft-2*) with the *bft* gene form more biofilms than the WT-NTBF without the *bft* gene. Nevertheless, our data showed that the *C. butyricum* CFS more effectively inhibited and eradicated biofilms of WT-ETBF (*bft-2*) and rETBF (*bft-2*) with the *bft* gene than WT-NTBF without the *bft* gene.

Furthermore, this study analyzed the expression levels of *ompA* and *bmeB3* genes according to the concentration of *C. butyricum* CFS by qRT-PCR. More specifically, the *ompA*, the most abundant outer membrane protein in *B. fragilis*, plays a structural role in capsule formation [48]. Lilian *et al*. have shown a positive correlation between increased adhesion and *ompA* gene expression in *B. fragilis*, suggesting that *ompA* gene was associated with adherence to human intestinal epithelial cells [48]. In addition, the *ompA* protein is involved in maintaining cell structure in the biofilms of *B. fragilis* [31]. Based on previous studies, it can be assumed that *ompA* gene plays a major role in the adhesion and biofilm formation of *B. fragilis*. Moreover, *B. fragilis* contains putative *luxR* orthologues that control biofilm formation, bmeB efflux pump expression, and susceptibility to antibiotics [49]. Also, the RND-type drug efflux pumps from gram-negative bacteria are related to virulence and biofilm formation [50]. This finding suggests that expression of *bmeB* gene related to RND-type efflux pump is associated with *B. fragilis* biofilm formation. Our study shows that the *C. butyricum* CFS inhibits biofilm formation of WT-ETBF and rETBF strains carrying the toxigenic *bft-2* gene by reducing the expression of virulence- and efflux pump-related genes (*ompA* and *bmeB3*) in *B. fragilis*. Our results indicated that *C. butyricum* CFS suppressed *ompA* and *bmeB3* genes only for *B. fragilis* strains with the *bft* gene.

Thus, our findings showed that CFS derived from *C. butyricum* exhibits antibacterial and antibiofilm activities against ETBF carrying the toxigenic *bft-2* gene by regulating the virulence- and efflux pump-related genes (*ompA* and *bmeB3*). This study demonstrates the effectiveness of CFS obtained from *C. butyricum* against biofilm formation and provides scientific evidence for the development of new antibacterial and antibiofilm agents. Furthermore, these results suggest the potential of *C. butyricum* CFS as biotherapeutic agent to prevent and treat IBD and/or CRC caused by ETBF. Since this study does not analyze the chemical composition of *C. butyricum* CFS, further study needs to thoroughly investigate the effects of the one major component in *C. butyricum* CFS against the growth and biofilm of ETBF. Although further studies are needed to determine its clinical application, the results suggest that *C. butyricum* acts as an effective probiotic against ETBF secreting Bft by reducing growth and biofilm formation.

## Figures and Tables

**Table 1:** Primer sequences used for qRT-PCR.

Name	Sequence (5’ → 3’)	Reference
*16S rRNA*	Forward AGTAGAGGTGGGCGGAATTC	[30]
	Reverse GTGTCAGTTGCAGTCCAGTG	
*ompA*	Forward GGATATGACGGTGTTGCCAG	[31]
	Reverse TAGCAGCAGCCATGTCATTC	
*bmeB3*	Forward GTACCGGAAGTTCAAGGTGT	[32]
	Reverse GAGCAGCCTCGATATTCTGT	
